# Rational Design of Chimeric Antisense Oligonucleotides on a Mixed PO–PS Backbone for Splice-Switching Applications

**DOI:** 10.3390/biom14070883

**Published:** 2024-07-22

**Authors:** Bao T. Le, Suxiang Chen, Rakesh N. Veedu

**Affiliations:** 1 Centre for Molecular Medicine and Innovative Therapeutics, Health Futures Institute, Murdoch University, Murdoch, WA 6150, Australia; tribaole@gmail.com (B.T.L.); suxiang.chen@murdoch.edu.au (S.C.); 2 ProGenis Pharmaceuticals Pty Ltd., Bentley, WA 6102, Australia; 3 Precision Nucleic Acid Therapeutics, Perron Institute for Neurological and Translational Science, Nedlands, WA 6009, Australia

**Keywords:** antisense oligonucleotide, splice-switching oligonucleotide, phosphodiester, phosphorothioate, binding affinity, exon skipping, nuclease stability

## Abstract

Synthetic antisense oligonucleotides (ASOs) are emerging as an attractive platform to treat various diseases. By specifically binding to a target mRNA transcript through Watson–Crick base pairing, ASOs can alter gene expression in a desirable fashion to either rescue loss of function or downregulate pathogenic protein expression. To be clinically relevant, ASOs are generally synthesized using modified analogs to enhance resistance to enzymatic degradation and pharmacokinetic and dynamic properties. Phosphorothioate (PS) belongs to the first generation of modified analogs and has played a vital role in the majority of approved ASO drugs, mainly based on the RNase H mechanism. In contrast to RNase H-dependent ASOs that bind and cleave target mature mRNA, splice-switching oligonucleotides (SSOs) mainly bind and alter precursor mRNA splicing in the cell nucleus. To date, only one approved SSO (Nusinersen) possesses a PS backbone. Typically, the synthesis of PS oligonucleotides generates two types of stereoisomers that could potentially impact the ASO’s pharmaco-properties. This can be limited by introducing the naturally occurring phosphodiester (PO) linkage to the ASO sequence. In this study, towards fine-tuning the current strategy in designing SSOs, we reported the design, synthesis, and evaluation of several stereo-random SSOs on a mixed PO–PS backbone for their binding affinity, biological potency, and nuclease stability. Based on the results, we propose that a combination of PO and PS linkages could represent a promising approach toward limiting undesirable stereoisomers while not largely compromising the efficacy of SSOs.

## 1. Introduction

Splice-switching oligonucleotides (SSOs) are a class of antisense oligonucleotides (ASOs) that can precisely target and modulate alternative splicing [1]. Unlike RNase H-dependent ASOs that bind and trigger the cleavage of target mature mRNA, SSOs mainly bind and block the access of splicing factors to the precursor mRNA in the cell nucleus [2,3]. By fine-tuning the design of SSOs, skipping or inclusion of an exon (or pseudoexon) can be achieved [1,2,3,4,5,6,7]. This approach has been successfully translated into therapeutic drugs to correct genetic mutation and restore functional protein expression. Upon the writing of this article, there are five SSO drugs (eteplirsen, nusinersen, golodirsen, viltolarsen, and casimersen) that have been approved by the US Food and Drug Administration (FDA) for the treatment of rare genetic disorders [8].

**Figure 1:**
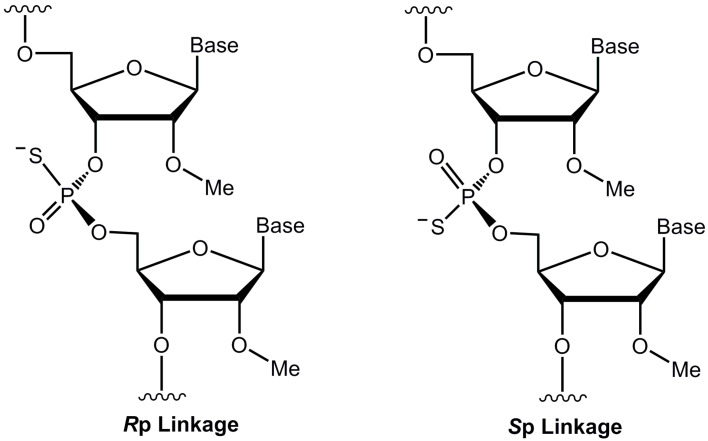
Structural representations of 2′-O-methyl (2′-OMe) RNA analog on phosphodiester (PO) or phosphorothioate (PS) backbone. (*R*p) and (*S*p) represent R and S stereoisomers, respectively.

The pharmaco-properties of SSOs, including pharmacology, pharmacokinetics, and pharmacodynamics, are solely dependent on their composition. Typically, SSOs are synthesized using modified nucleotide analogs to improve their stability and bio-distribution [9]. To date, one of the first and most widely used internucleotide linkage modifications is phosphorothioate (PS), where the conventional phosphodiester (PO) linkage is substituted with a PS group (Figure 1). The major advantage of a PS ASO over an unmodified one is enhanced nuclease stability, improving its half-life in circulation, and the ability to bind to various plasma proteins, which contributes to its enhanced bio-distribution [9,10].

One major limitation of PS ASOs compared with the unmodified ones arises from their stereochemical structures. Switching from a non-bridging oxygen atom to a sulfur atom introduces chirality to the structural configuration of PS ASOs, yielding two distinct classes of stereoisomers: ‘R’ and ‘S’ isomers (Figure 1) [9,10]. Although the stereoisomers generated during the synthesis of PS ASOs are still being investigated, the reported evidence indicates that stereoisomers do impact the performance of ASOs both in terms of toxicity and efficacy [10,11]. Various attempts have been made to synthesize and evaluate stereo-pure PS oligonucleotides; however, more data are required to draw more insights into the performance of stereo-pure variants [11,12,13]. One simple approach to improve any toxicities associated with PS linkages is to reduce the number of PS linkages within an oligonucleotide. This is the case with the recently approved drug eplontersen (AKCEA-TTR-L_Rx_) versus inotersen, where both drugs have identical base sequences, but eplontersen has six fewer PS linkages compared with inotersen [14]. A previously published study comparing these two drugs has shown that fewer PS linkages in ASOs could potentially reduce pro-inflammatory effects and result in similar or slightly better efficacy [15].

The synthesis of RNase H-dependent ASOs [12,16,17] and exon-skipping SSOs [18] with a mixed PO–PS backbone have recently been reported. However, the main focus of these efforts is to generate and evaluate stereo-pure oligonucleotides. In this study, using an established cellular model of Duchenne muscular dystrophy—*H-2K^b^*-tsA58 (H2K) *mdx* mouse myotubes [19,20]—we endeavor to explore different stereo-random SSO design modalities containing both PO and PS linkages. The SSOs were assessed based on their ability to induce exon skipping in the above-mentioned in vitro system.

## 2. Materials and Methods

### 2.1. Design and Synthesis of ASOs

In this study, we used *Dmd* M23D (+2–18), a previously reported 20mer ASO sequence designed to induce exon-23 skipping in mouse *Dmd* transcript [21,22,23,24,25,26], as a model sequence to incorporate our designs in which the internucleotide linkages (PO or PS) are distributed within the ASO sequence in the form of mixmer (alternating bases), gapmer, or fully modified. A fully 2′-O-methoxyethyl (2′-MOE)-PS-modified sequence designed to not target the dystrophin transcript was used as a negative control. All ASOs (Table 1) were synthesized by SynGenis Pty Ltd. (Bentley, WA, Australia).

### 2.2. Melting Temperature Study of ASOs

Six 2′-O-methyl (2′-OMe)-modified ASOs with a *Dmd* M23D (+2–18) sequence on various PO–PS backbones—5′-PO mixmer, 5′-PS mixmer, PO–PS–PO gapmer, PS–PO–PS gapmer, full PS, and full PO—were prepared at 2 µM concentration in a buffer solution containing 0.01 mM EDTA, 10 mM NaCl, and 10 mM sodium phosphate buffer (pH 7.0). Then, the ASOs were mixed with an equal volume of their complementary RNA sequence (5′-AGGUAAGCCGAGGUUUGGCC-3′) at the same concentration (2 µM) and denatured at 95 °C for 10 min, followed by gradual cooling to room temperature, and subsequently loaded onto quartz cuvettes of 1 mm path length. The melting temperature (T_m_) was analyzed on Shimadzu UV-1800 spectrophotometer (Rydalmere, NSW, Australia) with a temperature controller over the range of 20–95 °C (ramp rate = 1.0 °C/min). T_m_ values were calculated by the first derivative.

### 2.3. Cell Culture and Transfection of ASOs into Cells

Immortalized *H-2K^b^*-tsA58 (H2K) *mdx* mouse myoblasts were cultured and differentiated as described previously [19,20,21,22]. Briefly, H2K myoblasts were propagated in Dulbecco’s modified Eagle’s medium (DMEM) containing 20% fetal bovine serum (FBS), 10% horse serum (HS) supplemented with 0.5% chicken embryo extract (CEE) at 33 °C, 10% CO_2_. When the myoblasts reached 60–90% confluency, they were trypsinized and seeded into 24-well plates (2 × 10^4^ cells/well) pre-treated with 50 μg/mL poly-D-lysine (Sigma Aldrich; Castle Hill, NSW, Australia) and 100 μg/mL Matrigel (Corning, supplied through In Vitro Technologies, Noble Park North, VIC, Australia). The cells were differentiated into myotubes at 37 °C, 5% CO_2_ in low glucose DMEM containing 5% HS for 24 h. For transfection, ASOs were complexed with Lipofectin reagent (Life Technologies, Carlsbad, CA, USA) at the ratio of 2:1 (lipofectin:ASO) with final concentrations at 400, 200, and 100 nM in a volume of 500 μL/well in 24-well plates, as per the manufacturer’s instructions, except that the medium was not removed after 3 h.

### 2.4. RNA Extraction and Reverse Transcription-Polymerase Chain Reaction (RT-PCR)

Twenty-four hours after transfection, cells were collected, and RNA extraction was subsequently performed using the ISOLATE II RNA Mini Kit (Bioline, Eveleigh, NSW, Australia) as per the manufacturer’s instructions. The dystrophin transcripts were amplified by nested RT-PCR across exons 20–26 using SuperScript™ III Reverse Transcriptase (for primary amplification) and AmpliTaq Gold™ 360 DNA Polymerase (for secondary PCR) as described previously [21,22]. The primer sets and PCR conditions used for the two-step reactions are shown in Table S1 (Supplementary Information). The secondary PCR products were separated on 2% agarose gels in Tris–acetate–EDTA buffer, and images were captured on a Fusion Fx gel documentation system (Vilber Lourmat, Marne-la-Vallee, France). Densitometry analysis of the bands was performed using ImageJ software [27] with both original gel images.

### 2.5. Nuclease Stability Study of ASOs

The stability of ASOs against 3′ → 5′ exonuclease degradation was evaluated using snake venom phosphodiesterase (Sigma Aldrich; Bayswater, VIC, Australia). Briefly, 10 μM of ASOs was mixed with phosphodiesterase (final concentration: 0.004 unit/μL) in a buffer of 10 mM Tris–HCl, 100 mM NaCl, and 15 mM MgCl_2_. The mixtures were incubated at 37 °C, and aliquots were collected at different time points and then quenched with an equal volume of 80% formamide containing bromophenol blue and xylene cyanol gel tracking dyes, followed by heating at 95 °C for 5 min. The quenched samples were then analyzed using 20% denaturing polyacrylamide gel electrophoresis. Images were captured on a Fusion Fx gel documentation system (Vilber Lourmat, Marne-la-Vallee, France).

## 3. Results

In this study, we utilized a previously reported ASO sequence *Dmd* M23D (+2–18) as a model to explore different design modalities on a mixed PO-PS backbone. All ASOs were synthesized using 2′-OMe chemistry (one of the standard modifications on sugar-moiety in SSOs) in which the internucleotide linkages were incorporated in the form of PO–PS mixmers (5′-PO and 5′-PS), gapmers (PO–PS–PO and PS–PO–PS), or fully modified (full PS and full PO) (Table 1). The fully PS-modified ASO was used as a positive control. The ASOs were assessed based on their binding affinity via melting temperature studies, their biological potency via exon-skipping experiments, and their stability against 3′ → 5′ exonuclease via nuclease degradation assays.

### 3.1. Evaluation of RNA Binding Affinity of ASOs

In order to assess the *Dmd* transcript binding affinity of different PO–PS ASOs, a thermal stability study of the ASO sequences against their complementary synthetic RNA sequence was performed. The results showed that the full-PO ASO exhibited the highest T_m_ (67.9 °C) while its full-PS counterpart displayed the lowest melting temperature (63.8 °C). Although all the PO–PS chimeric ASOs (including mixmers and gapmers) contain around half the number of PS linkages in the full-PS oligonucleotide, their T_m_ values were only slightly lower than (5′-PO mixmer: 67.5 °C, PO–PS–PO gapmer: 66.3 °C, PS–PO–PS gapmer: 66.7 °C) or even identical to (5′-PS mixmer: 67.9 °C) the full-PO ASO (Table 2). Interestingly, the T_m_ values of both mixmers (5′-PO: 67.5 °C, 5′-PS: 67.9 °C) are higher than the gapmers (PO–PS–PO: 66.3 °C, PS–PO–PS: 66.7 °C), and the ASOs having PS linkages on both flanks (5′-PS mixmer: 67.9 °C, PS–PO–PS gapmer: 66.7 °C) exhibited somewhat higher T_m_ than their counterparts with flanking PO linkages (5′-PO mixmer: 67.5 °C, PO–PS–PO gapmer: 66.3 °C, respectively). 

### 3.2. Evaluation of ASOs in Inducing Exon Skipping of Dystrophin Transcript In Vitro

In an attempt to investigate the splice-switching capability of PO–PS ASOs in vitro, the exon-skipping assay was performed in duplicates by transfecting the ASOs (400, 200, and 100 nM) to H2K *mdx* myotubes. After 24 h incubation, cells were collected, and RNA was extracted and amplified using the RT-PCR protocol reported previously [21,22,23,24,25,26]. The exon-skipping efficiency was determined by the percentages of PCR products amplified by the exon-23 skipping (688 bp) and exon-22/23 dual-skipping (542 bp) over the full-length (901 bp).

**Figure 2:**
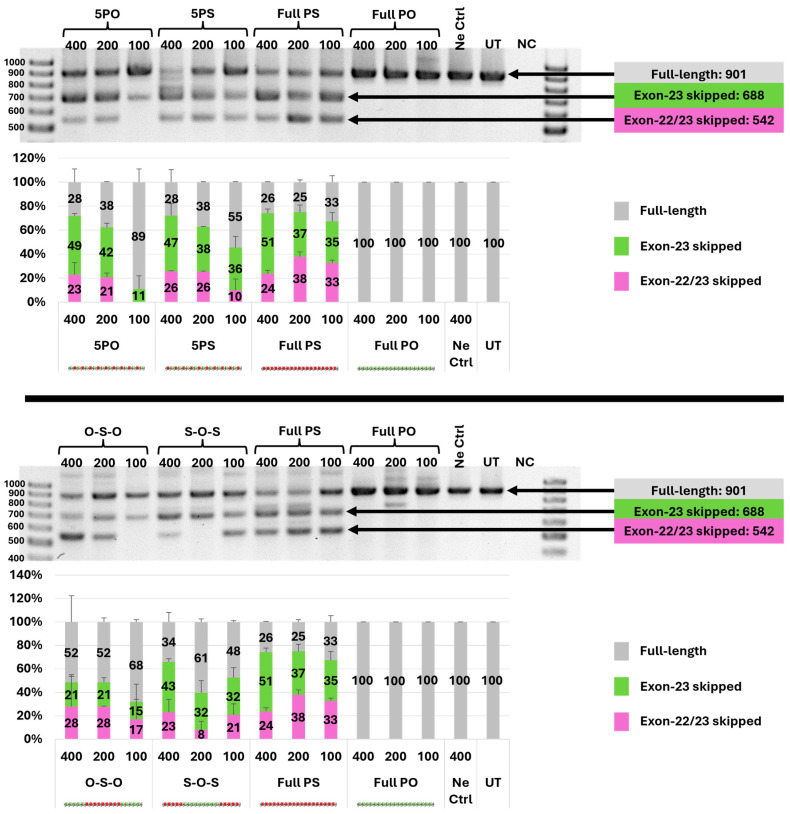
Representative agarose gel images and densitometry analysis (in duplicates) of RT-PCR products amplified from RNA extracted from cells transfected with ASO concentrations at 400, 200, and 100 nM. The original gel images are shown in Figure S2 (Supplementary Information). Ne Ctrl: negative control sequence; UT: untreated; NC: PCR sample with no added RNA template.

In general, the results clearly demonstrated that the fully PS-modified ASO (positive control) induced exon skipping in the dystrophin transcript more efficiently than all chimeric PO–PS ASOs, while the full-PO ASO did not cause any exon skipping (Figure 2 and Figure S2). Typically, at 400 nM concentration, the full-PS ASO exhibited higher exon-23 skipping (51%) than both the mixmers (5′-PO: 49%, 5′-PS: 47%) and gapmers (PO–PS–PO: 21%, PS–PO–PS: 43%). Among the chimeric PO–PS ASOs, it is obvious that the mixmers were more efficient at inducing exon skipping than the PO–PS–PO gapmer in most cases (Figure 2 and Figure S2). More details, at 200 nM concentration, the mixmers achieved significantly higher exon-23 skipping (5′-PO: 42%, 5′-PS: 38%) than the PO–PS–PO gapmer (21%). To gain more insights into the effect of the positioning of PS linkages in each design, we further compared the exon-skipping efficiency between different designs of mixmers and gapmers. Regarding the two mixmers, 5′-PS displayed comparable exon-skipping efficiency with its 5′-PO counterpart at 400 and 200 nM concentrations, while at 100 nM, 5′-PS induced higher exon-23 skipping than the 5′-PO ASO (5′-PS: 36%, 5′-PO: 11%). For the two gapmers, PS–PO–PS clearly showed better exon-23 skipping ability than its PO–PS–PO counterpart at all concentrations (PS–PO–PS: 43% at 400 nM, 32% at 200 and 100 nM; PO–PS–PO: 21% at 400 and 200 nM, 15% at 100 nM).

### 3.3. Evaluation of Nuclease Stability of ASOs

**Figure 3:**
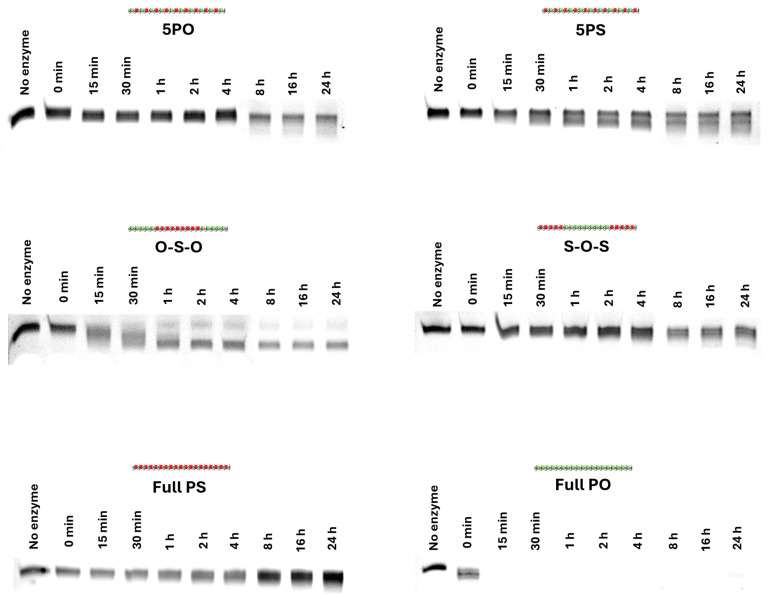
Polyacrylamide gel images of the nuclease stability assay. Time points include 0, 0.25, 0.5, 1, 2, 4, 8, 16, and 24 h. The original gel images are shown in Figure S3 (Supplementary Information).

In order to further characterize the PO–PS ASOs, the nuclease stability of the ASOs was investigated in a systematic manner by co-incubation of the ASOs with snake venom phosphodiesterase for short (15 min, 30 min, 1 h, 2 h, 4 h) and longer (8 h, 16 h, 24 h) periods of time. Firstly, the full-PS, full-PO, and chimeric PO–PS ASOs were incubated with phosphodiesterase I (possessing high 3′ → 5′ exonuclease activity) from the venom of eastern diamondback rattlesnake (*Crotalus adamanteus*) at 37 °C for 15 min (0.25 h), 30 min (0.5 h), 1 h, 2 h, and 4 h. For these short periods, the full-PS ASO exhibited higher nuclease resistance than other oligonucleotides, while the full-PO ASO did not show any resistance against nuclease that it was completely degraded within 15 min (Figure 3 and Figure S3). Interestingly, the 5′-PO mixmer, 5′-PS mixmer, and PS–PO–PS gapmer were more resistant to nuclease than the PO–PS–PO gapmer (Figure 3 and Figure S3). To gain more insights into the prolonged stability of the ASOs, a longer incubation (8 h, 16 h, and 24 h) experiment was performed. The results confirmed that full-PS modification remained the most stable backbone design compared with other candidates, while the PO–PS–PO design was less resistant to nuclease than other chimeras (Figure 3 and Figure S3). Remarkably, the 5′-PO mixmer, 5′-PS mixmer, and PS–PO–PS gapmer exhibited comparable stability after being treated with nuclease for 24 h.

## 4. Discussion

Modified nucleic acid analogs play a vital role in any successful translation of ASO therapeutics. Among hundreds of modifications described in the literature, phosphorothioate (PS) is regarded as one of the earliest and most widely used linkages in RNA drug development [9,10]. Eckstein and colleagues first reported the synthesis of a PS oligonucleotide in 1969 with better resistance to enzymatic degradation and biological activity [28]. PS modification has now been utilized in antisense research for over 40 years, and the majority of the FDA-approved ASO drugs possess a PS backbone, mainly based on the RNase H mechanism [3]. PS ASOs show enhanced resistance to nucleases and better bio-distribution profiles than the unmodified linkages. However, the stereochemistry of PS oligonucleotides has been a limitation in relation to its efficacy and toxicity. In a quest to reduce the number of undesirable stereoisomers generated during the synthesis of PS ASOs, in this study, we evaluated the effect of reduced PS linkages within ASO sequence by synthesizing PO–PS mixmers and gapmers and comparing them with full-PS and full-PO controls.

Towards the ASO performance, we first assessed its binding affinity by performing a melting temperature study against a synthetic complementary RNA sequence. The T*_m_* order was revealed as follows: full PO (67.9 °C) = 5′-PS (67.9 °C) > 5′-PO (67.5 °C) > PS–PO–PS (66.6 °C) > PO–PS–PO (66.3 °C) > full PS (63.8 °C). Surprisingly, the 5′-PS has an identical T*_m_* to the full PO while possessing ten PS linkages compared with none in the full PO. This is unexpected as it is well-known that PS linkage destabilizes RNA duplex, which should result in lower T*_m_*. In our opinion, the position of PS linkage is crucial to this observation, as per the cases of 5′-PO versus 5′-PS and PO–PS–PO versus PS–PO–PS. In fact, in both cases, having one more PS linkage (5′-PS and PS–PO–PS) increases the T*_m_* by 0.4 °C (rather than decreasing). Interestingly, since Rp linkages have been reported to be more thermally stable than Sp linkages [12], we might be seeing a mixed effect from a stereo-random sequence. Overall, the results of the melting temperature studies indicate that the rational design of chimeric PO–PS ASOs could not only reduce the number of PS linkages in a full-PS ASO sequence but also largely maintain the high RNA binding affinity of full-PO ASO.

Next, we evaluated the efficacy of the ASOs in an exon-skipping assay using a well-known DMD in vitro model H2K *mdx* myotubes. This model has been utilized in our laboratory as a validated biological system to test numerous chemically modified ASOs [26]. The results indicated that, in terms of inducing exon skipping, 5′-PS and PS–PO–PS are the better performers between the mixmers and the gapmers, respectively. This is encouraging as the 5′-PS and PS–PO–PS possess only 10 PS linkages, compared with 19 PS linkages in the full PS, which translates to a significant reduction in stereoisomers. Interestingly, at 200 nM, PS–PO–PS gapmer showed less efficient exon-skipping activity both than 100 nM and 400 nM, indicating that the dose dependency of chimeric oligomers might not be as consistent as their counterparts of uniform chemistry (full PS and full PO). We also noted some inconsistencies with the PO–PS–PO at 100 nM concentration, which could be due to its enzymatic degradation.

In addition, we also investigated the nuclease stability of the chimeric ASOs against a harsh 3′ → 5′ exonuclease. The results demonstrated that the full PS is the most stable ASO, opposite to the full PO, which was completely degraded after 15 min of incubation. This is in line with our observation in the exon-skipping assay, as the full PO did not induce any exon skipping, whereas the full PS was very efficient. In comparison to other chimeric ASOs, PO–PS–PO is the least stable. This could contribute to our inconsistent results observed in the exon-skipping assay. On the other hand, the other three chimeric ASOs (5′-PO, 5′-PS, and PS–PO–PS) showed better resistance to nuclease.

## 5. Conclusions

Reducing the number of PS linkages within a therapeutic ASO sequence presents a potential strategy to mitigate the impact of PS stereoisomers on their drug-like properties. To optimize the design strategy for splice-switching ASOs (i.e., SSOs) with reduced PS linkages, we designed different mixmer-like and gapmer-like chimeric PO–PS ASOs and investigated the performance in terms of target binding affinity, splice-switching capability, and nuclease stability. We clearly found that not only the quantity of PS linkages but also their positioning in an ASO sequence plays an essential role in the ASO’s potency. Based on the results, we believe that chimeric PO–PS design is a useful strategy for reducing the generation of PS stereoisomers and placing PS linkages at both the 5′-end and the 3′-end might potentially retain the high target binding affinity and nuclease stability while not substantially compromising the exon-skipping efficacy.

## Figures and Tables

**Table 1:** ASO sequences used in this study.

Sequence Name	Backbone Composition			ASO Sequence (5′-3′)	Abbreviation
*Dmd* M23D (+2–18)	Full 2′-OMe	Full PO		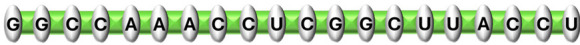	Full PO
		Full PS		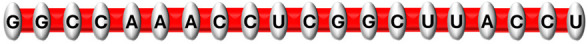	Full PS
		PO–PS mixmers	5′-PO	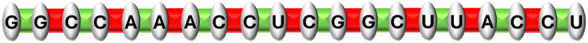	5PO
			5′-PS	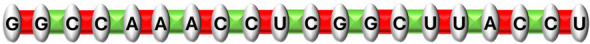	5PS
		PO–PS gapmers	PO–PS–PO	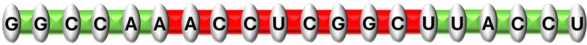	O-S-O
			PS–PO–PS	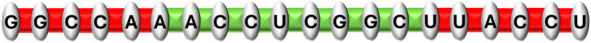	S-O-S
Negative control	Full 2′-MOE-PS			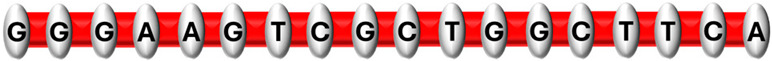	Ne Ctrl

PO linkages are represented as green color bars; PS linkages are represented as red color bars. ASO: antisense oligonucleotide; 2′-OMe: 2′-O-methyl; 2′-MOE: 2′-O-methoxyethyl; PO: phosphodiester; PS: phosphorothioate; Ne Ctrl: negative control sequence.

**Table 2:** ASO sequences used in this study and their melting temperature.

PO–PS Designs of 2′-OMe-Modified *Dmd* M23D (+2–18)	Number of PS Linkages	T*_m_*, °C
5′-PO mixmer	9	67.5
5′-PS mixmer	10	67.9
PO–PS–PO gapmer	9	66.3
PS–PO–PS gapmer	10	66.7
Full PS	19	63.8
Full PO	0	67.9

2′-OMe: 2′-O-methy; PO: phosphodiester; PS: phosphorothioate; T_m_: melting temperature. Please see Figure S1 (Supplementary Information) for melting curves.

## Data Availability

The data used in this study are available from the corresponding author upon reasonable request.

## Supplementary Materials

The following supporting information can be downloaded at https://www.mdpi.com/article/10.3390/biom14070883/s1, Figure S1: Melting profiles of the ASOs against a synthetic complementary RNA; Figure S2: Original agarose gel images of RT-PCR products (in duplicates); Figure S3: Original polyacrylamide gel images of the nuclease stability assay; Table S1: The primer sequences and PCR conditions used for the amplification of dystrophin transcripts.

## References

[1] Havens M.A., Hastings M.L. (2016). Splice-switching Antisense Oligonucleotides as Therapeutic Drugs. Nucleic Acids Res..

[2] Dias N., Stein C.A. (2002). Antisense Oligonucleotides: Basic Concepts and Mechanisms. Mol. Cancer Ther..

[3] Crooke S.T., Liang X.H., Baker B.F., Crooke R.M. (2021). Antisense Technology: A Review. J. Biol. Chem..

[4] Le B.T., Chen S., Veedu R.N. (2023). Evaluation of Chemically Modified Nucleic Acid Analogues for Splice Switching Application. ACS Omega.

[5] Chen S., Heendeniya S.N., Le B.T., Rahimizadeh K., Rabiee N., Zahra Q.U.A., Veedu R.N. (2024). Splice-Modulating Antisense Oligonucleotides as Therapeutics for Inherited Metabolic Diseases. BioDrugs.

[6] Li Y., Tan Y., Zhang R., Wang T., Na N., Zheng T., Veedu R.N., Chen S. (2022). Antisense Oligonucleotide: A Potential Therapeutic Intervention for Chronic Kidney Disease. Kidney Dial..

[7] Chen S., Sbuh N., Veedu R.N. (2021). Antisense Oligonucleotides as Potential Therapeutics for Type 2 Diabetes. Nucleic Acid Ther..

[8] Thakur S., Sinhari A., Jain P., Jadhav H.R.A. (2022). Perspective on Oligonucleotide Therapy: Approaches to Patient Customization. Front. Pharmacol..

[9] Agrawal S. (2021). The Evolution of Antisense Oligonucleotide Chemistry—A Personal Journey. Biomedicines.

[10] Crooke S.T., Vickers T.A., Liang X.H. (2020). Phosphorothioate Modified Oligonucleotide-protein Interactions. Nucleic Acids Res..

[11] Wan W.B., Migawa M.T., Vasquez G., Murray H.M., Nichols J.G., Gaus H., Berdeja A., Lee S., Hart C.E., Lima W.F. (2014). Synthesis, Biophysical Properties and Biological Activity of Second Generation Antisense Oligonucleotides containing Chiral Phosphorothioate Linkages. Nucleic Acids Res..

[12] Iwamoto N., Butler D.C.D., Svrzikapa N., Mohapatra S., Zlatev I., Sah D.W.Y., Meena, Standley S.M., Lu G., Apponi L.H. (2017). Control of Phosphorothioate Stereochemistry Substantially Increases the Efficacy of Antisense Oligonucleotides. Nat. Biotechnol..

[13] Li M., Lightfoot H.L., Halloy F., Malinowska A.L., Berk C., Behera A., Schümperli D., Hall J. (2017). Synthesis and Cellular Activity of Stereochemically-pure 2′-O-(2-methoxyethyl)-Phosphorothioate Oligonucleotides. Chem. Commun..

[14] Viney N.J., Guo S., Tai L.J., Baker B.F., Aghajan M., Jung S.W., Yu R.Z., Booten S., Murray H., Machemer T. (2021). Ligand Conjugated Antisense Oligonucleotide for the Treatment of Transthyretin Amyloidosis: Preclinical and Phase 1 Data. ESC Heart Fail..

[15] Prakash T.P., Yu J., Migawa M.T., Kinberger G.A., Wan W.B., Østergaard M.E., Carty R.L., Vasquez G., Low A., Chappell A. (2016). Comprehensive Structure-Activity Relationship of Triantennary N-Acetylgalactosamine Conjugated Antisense Oligonucleotides for Targeted Delivery to Hepatocytes. J. Med. Chem..

[16] Byrne M., Vathipadiekal V., Apponi L., Iwamoto N., Kandasamy P., Longo K., Liu F., Looby R., Norwood L., Shah A. (2021). Stereochemistry Enhances Potency, Efficacy, and Durability of *Malat1* Antisense Oligonucleotides In Vitro and In Vivo in Multiple Species. Transl. Vis. Sci. Technol..

[17] Liu Y., Dodart J.C., Tran H., Berkovitch S., Braun M., Byrne M., Durbin A.F., Hu X.S., Iwamoto N., Jang H.G. (2021). Variant-selective Stereopure Oligonucleotides Protect against Pathologies Associated with C9orf72-repeat Expansion in Preclinical Models. Nat. Commun..

[18] Kandasamy P., McClorey G., Shimizu M., Kothari N., Alam R., Iwamoto N., Kumarasamy J., Bommineni G.R., Bezigian A., Chivatakarn O. (2022). Control of Backbone Chemistry and Chirality Boost Oligonucleotide Splice Switching Activity. Nucleic Acids Res..

[19] Bulfield G., Siller W.G., Wight P.A., Moore K.J. (1984). X Chromosome-linked Muscular Dystrophy (*mdx*) in the mouse. Proc. Natl. Acad. Sci. USA.

[20] Rando T.A., Blau H.M. (1994). Primary Mouse Myoblast Purification, Characterization, and Transplantation for Cell-mediated Gene Therapy. J. Cell Biol..

[21] Wilton S.D., Lloyd F., Carville K., Fletcher S., Honeyman K., Agrawal S., Kole R. (1999). Specific Removal of the Nonsense Mutation from the *mdx* Dystrophin mRNA Using Antisense Oligonucleotides. Neuromuscul. Disord..

[22] Mann C.J., Honeyman K., Cheng A.J., Ly T., Lloyd F., Fletcher S., Morgan J.E., Partridge T.A., Wilton S.D. (2001). Antisense-induced Exon Skipping and Synthesis of Dystrophin in the *mdx* Mouse. Proc. Natl. Acad. Sci. USA.

[23] Le B.T., Paul S., Jastrzebska K., Langer H., Caruthers M.H., Veedu R.N. (2022). Thiomorpholino Oligonucleotides as a Robust Class of Next Generation Platforms for Alternate mRNA Splicing. Proc. Natl. Acad. Sci. USA.

[24] Chen S., Le B.T., Chakravarthy M., Kosbar T.R., Veedu R.N. (2019). Systematic Evaluation of 2′-Fluoro Modified Chimeric Antisense Oligonucleotide-mediated Exon Skipping In Vitro. Sci. Rep..

[25] Le B.T., Adams A.M., Fletcher S., Wilton S.D., Veedu R.N. (2017). Rational Design of Short Locked Nucleic Acid-Modified 2′-O-Methyl Antisense Oligonucleotides for Efficient Exon-Skipping In Vitro. Mol. Ther. Nucleic Acids.

[26] Le B.T., Chen S., Abramov M., Herdewijn P., Veedu R.N. (2016). Evaluation of Anhydrohexitol Nucleic Acid, Cyclohexenyl Nucleic Acid and _D_-altritol Nucleic Acid-modified 2′-O-methyl RNA Mixmer Antisense Oligonucleotides for Exon Skipping In Vitro. Chem. Commun..

[27] Schneider C.A., Rasband W.S., Eliceiri K.W. (2012). NIH Image to ImageJ: 25 Years of Image Analysis. Nat Methods.

[28] De Clercq E., Eckstein E., Merigan T.C. (1969). Interferon Induction Increased through Chemical Modification of a Synthetic Polyribonucleotide. Science.

