# Supplementary material for: Rational Design of Chimeric Antisense Oligonucleotides on a Mixed PO–PS Backbone for Splice-Switching Applications
Source: Biomolecules. 2024 Jul 22;14(7):883. doi: 10.3390/biom14070883 (PMC11274656; doi:10.3390/biom14070883)
Supplement: Supplementary file 1 [file biomolecules-14-00883-s001.zip › biomolecules-3094956-supplementary.pdf]

Supplementary Information

Rational design of chimeric antisense oligonucleotides on a mixed PO-PS backbone for splice switching applications

Bao T. Le [1,2,†], Suxiang Chen [1,3,†] and Rakesh N. Veedu [1,2,3*]

[1]Centre for Molecular Medicine and Innovative Therapeutics, Health Futures Institute, Murdoch University, Murdoch, WA, 6150, Australia

[2]ProGenis Pharmaceuticals Pty Ltd, Bentley, WA, 6102, Australia

[3] Precision Nucleic Acid Therapeutics, Perron Institute for Neurological and Translational Science, Nedlands, WA, 6009, Australia

***Correspondence: R.Veedu@murdoch.edu.au**

† These authors contributed equally

Table S1. The primer sequences and PCR conditions used for the amplification of dystrophin transcripts.

Primary PCR		
Primer set	Ex20Fo	5'-CAGAATTCTGCCAATTGCTGAG-3'
	Ex26Ro	5'-TTCTTCAGCTTGTGTCATCC-3'
PCR conditions	55 °C for 30 min, 94 °C for 2 min before entering 31 cycles of 94 °C for 30 s, 55 °C for 30 s and 68 °C for 90 s	
**Secondary PCR**		
Primer set	Ex20Fi	5'-CCCAGTCTACCACCCTATCAGAGC-3'
	Ex26Ri	5'-CCTGCCTTTAAGGCTTCCTT-3'
PCR conditions	94 °C for 6 min before entering 33 cycles of 94 °C for 30 s, 55 °C for 1 min and 72 °C for 2 min	

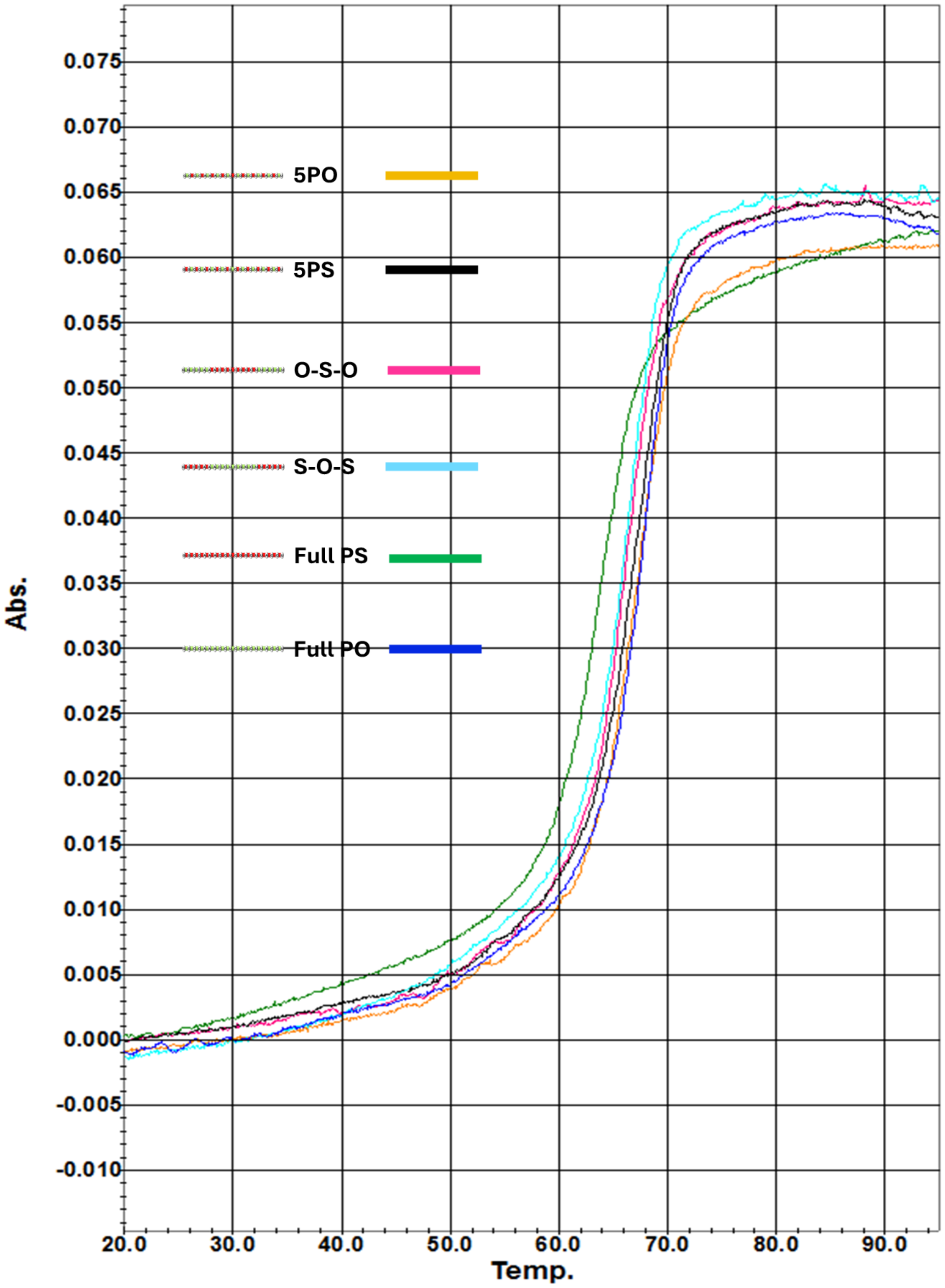

Figure S1. Melting profiles of the ASOs against a synthetic complementary RNA.

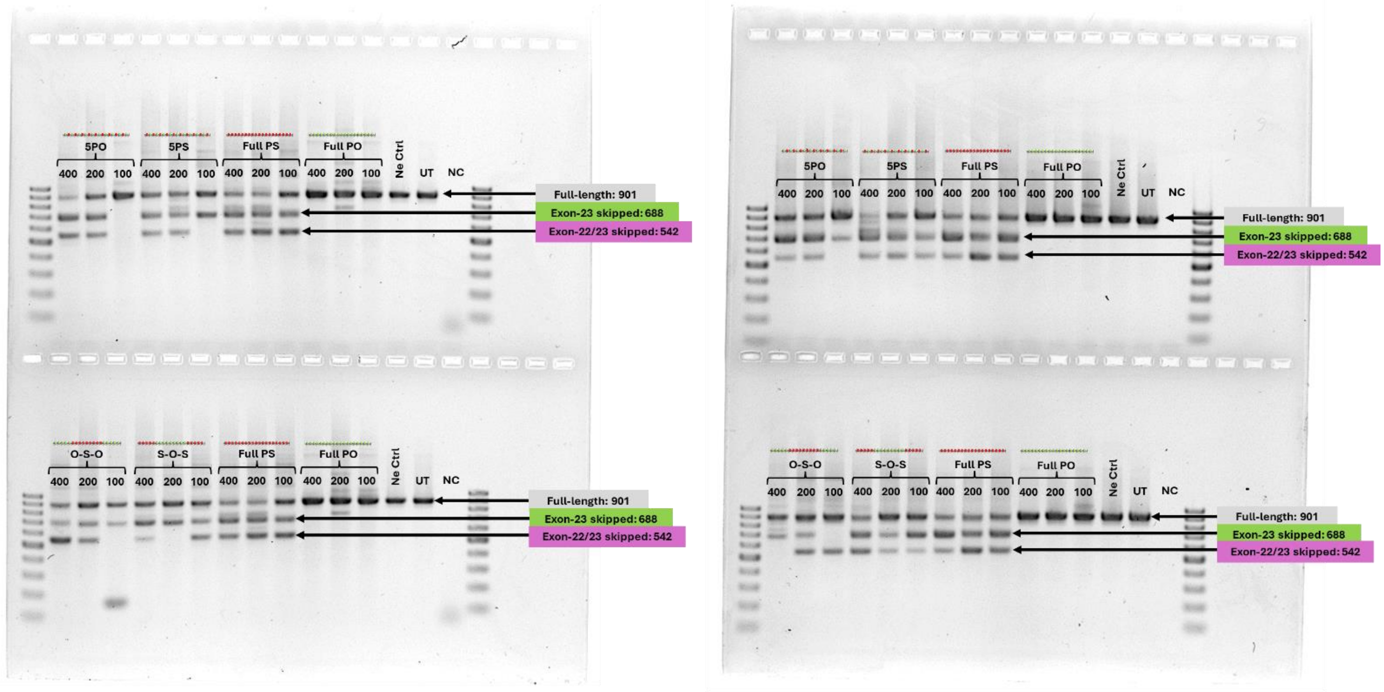

Repetition 1 **Repetition 2**

Figure S2. Original agarose gel images of RT-PCR products (in duplicates).

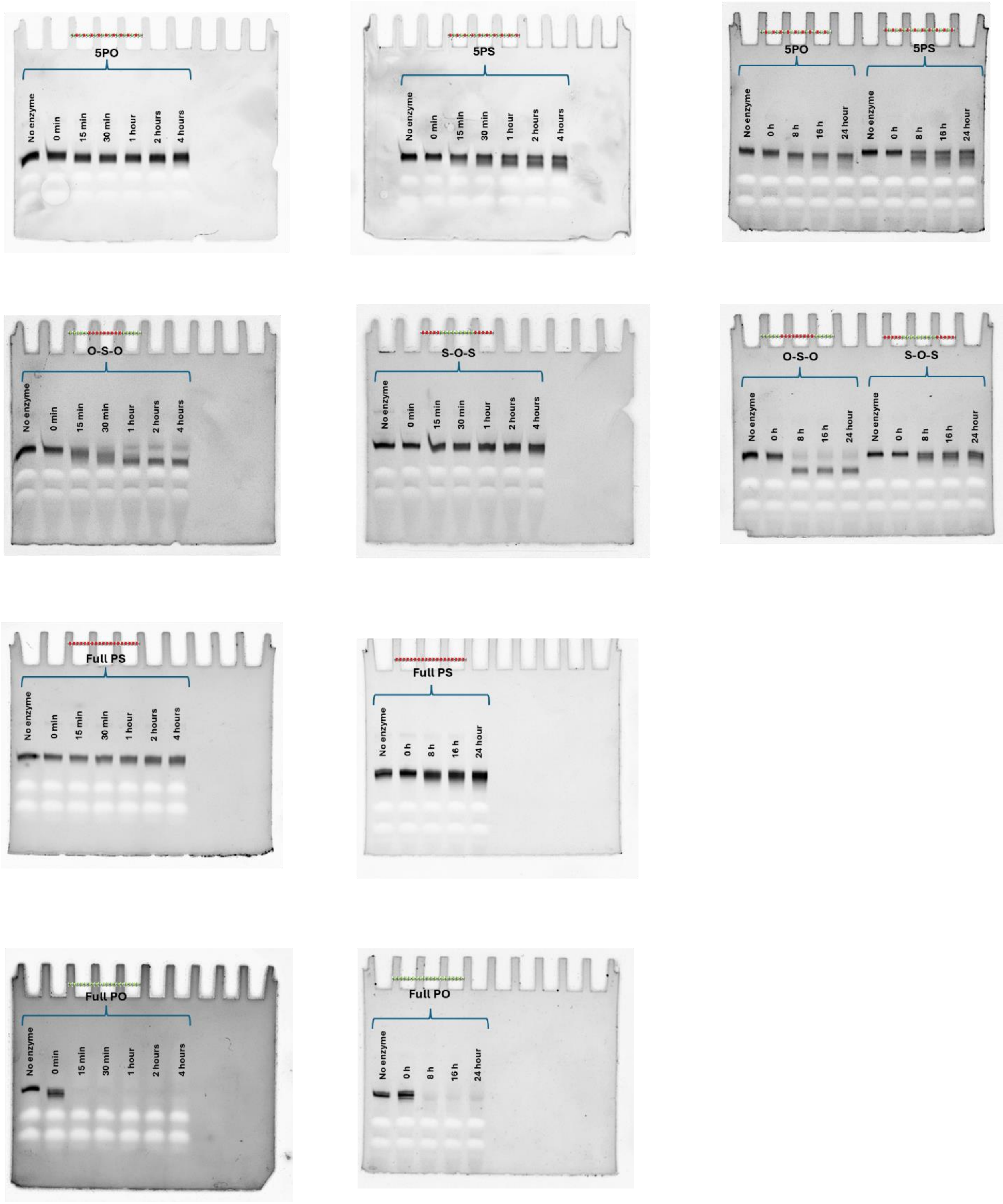

Figure S3. Original polyacrylamide gel images of the nuclease stability assay.
